# HIDDEN BARRIERS TO ENGAGING HOME- AND COMMUNITY-BASED SERVICES IN DEMENTIA CARE RESEARCH

**DOI:** 10.1093/geroni/igad104.0526

**Published:** 2023-12-21

**Authors:** Lauren Parker, Katherine Marx, Joseph Gaugler, Laura Gitlin

**Affiliations:** Johns Hopkins University, Baltimore, Maryland, United States; Johns Hopkins University, Baltimore, Maryland, United States; University of Minnesota, Minneapolis, Minnesota, United States; Drexel University, Philadelphia, Pennsylvania, United States

## Abstract

The high prevalence of dementia in the United States has resulted in a reliance on home-and community-based services (HCBS) to assist in the provision of care for community-dwelling persons living with dementia. Partnering with HCBS organizations, like Adult Day Services (ADS), is beneficial to disseminating evidence-based dementia care interventions as it is an existing service that can be leveraged to foster relationships between research staff and clients. While benefits exist, challenges in identifying community-partner organization, ethical approval, and community-partner staffing can be unintended barriers. Using the ADS Plus Program as a case-study, this presentation will detail hidden barriers in engaging HCBS partnerships into dementia care research. Specifically, we will discuss barriers to 1) developing relationships to implement a multi-site national-trial, 2) high touch points necessary to engage sites from underrepresented populations, 3) institutional review board requirements (e.g. CiTi training, Federal Wide Assurance Forms), and 4) staff readiness to participate in research. We also offer recommendations based on lessons learned from the ADS Plus Program to enhance the pipeline from engagement with HCBS to participate in research to implementing an evidence-based interventions. Lessons learned from the ADS Plus Program demonstrates that changes are needed to improve the efficiency of the pipeline from community-based participation into dementia care research and implementation of evidence-based interventions.

